# Post-hypoxia Invasion of the fetal brain by multidrug resistant *Staphylococcus*

**DOI:** 10.1038/s41598-017-06789-6

**Published:** 2017-07-25

**Authors:** Miguel A. Zarate, Michelle D. Rodriguez, Eileen I. Chang, Jordan T. Russell, Thomas J. Arndt, Elaine M. Richards, Beronica A. Ocasio, Eva Aranda, Elizabeth M. Gordon, Kevin Yu, Josef Neu, Maureen Keller-Wood, Eric W. Triplett, Charles E. Wood

**Affiliations:** 1 Department of Physiology and Functional Genomics, University of Florida College of Medicine, Gainesville, Florida USA; 2 Department of Microbiology and Cell Science, Institute of Food and Agricultural Sciences, University of Florida, Gainesville, Florida USA; 3 Department of Pharmacodynamics, University of Florida College of Pharmacy, Gainesville, Florida USA; 4 Department of Pediatrics, University of Florida College of Medicine, Gainesville, Florida USA

## Abstract

Herein we describe an association between activation of inflammatory pathways following transient hypoxia and the appearance of the multidrug resistant bacteria *Staphylococcus simulans* in the fetal brain. Reduction of maternal arterial oxygen tension by 50% over 30 min resulted in a subseiuent significant over-expression of genes associated with immune responses 24 h later in the fetal brain. The activated genes were consistent with stimulation by bacterial lipopolysaccharide; an influx of macrophages and appearance of live bacteria were found in these fetal brains. *S. simulans* was the predominant bacterial species in fetal brain after hypoxia, but was found in placenta of all animals. Strains of *S. simulans* from the placenta and fetal brain were equally highly resistant to multiple antibiotics including methicillin and had identical genome sequences. These results suggest that bacteria from the placenta invade the fetal brain after maternal hypoxia.

## Introduction

Transient fetal hypoxia is a common homeostatic disturbance during fetal life. Any compromise of maternal oxygen supply, for example high altitude or maternal hypoventilation secondary to any cause, decreases oxygen in the fetal blood. The fetal response to hypoxia is a homeostatic cardiovascular response that spares brain, adrenal, and heart viability. Cardiovascular and neuroendocrine reflex responses to hypoxia redirect blood flow such that a larger proportion of the combined ventricular output flows towards the brain, heart and adrenal glands^1^.

A consequence of severe or prolonged fetal hypoxia is brain cellular damage, thought to be due to oxygen starvation and a secondary inflammatory response^2, 3^. Less is known about the cellular response to hypoxia of short duration. We have found that the fetal cerebral cortex responds to 30 min of transient hypoxia with decreased expression of genes within metabolic pathways, and increased expression of genes within inflammatory pathways^4^. While much of our work to date has focused on the acute responses, systems modeling of the delayed (24 hr) responses to hypoxia in both cerebral cortex and kidney cortex have revealed significant upregulation of pathways involved in inflammation, including much of the inflammatory cascade, from toll-like receptors (TLR) to interleukin 1β^4^. Thus, the transcriptomics strongly suggested infiltration of these brain regions with macrophages or microglia. Herein we test the hypotheses that the hippocampus and hypothalamus would demonstrate similar responses to hypoxia, involving the entire inflammatory cascade and stimulating the influx of macrophages or microglia. We further hypothesized that based on the known ligand-receptor interactions for TLR2 and TLR4, a microbial exposure likely exists in the brain of hypoxia-treated fetuses. The results reported here confirm presence of macrophages and bacteria in the fetal brain 24 h after transient maternal and fetal hypoxia; the identity of that microbe, and its possible route of entry into the fetus, is described.

## Induction of inflammatory pathways in fetal brain

We expanded the investigation of the effects of maternal hypoxia on fetal responses by inclusion of hypothalamus and hippocampus. As in the previous report the tissues were obtained 24 h after a 30-minute period to hypoxia in pregnant ewes^4, 5^. Maternal and fetal arterial oxygen tensions were reduced from 98 ± 2 to 56 ± 2 and from 17 ± 1 to 10 ± 1 mm Hg, respectively. This is a severity of hypoxia similar to that observed by acute exposure to approximately 3600 m elevation^6^.

**Figure 1:**
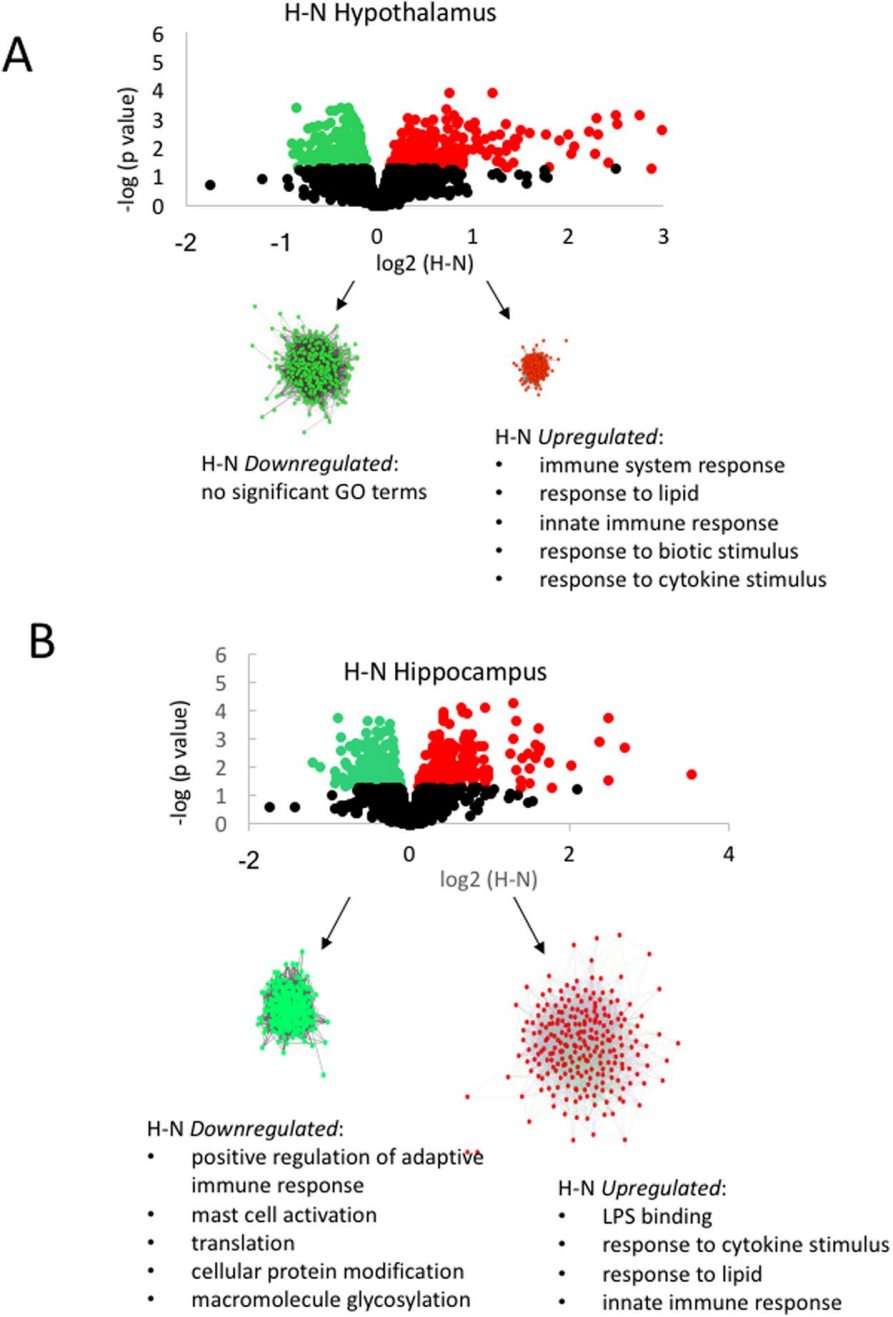
Panel A, analysis of gene expression in fetal hypothalamus using Agilent ovine 15.5k array revealed significant up- and down-regulation of gene expression by hypoxia compared to normoxia (H-N). Network inference and statistical modeling of gene ontology terms revealed that hypoxia upregulated pathways related to inflammation. Panel B, significant up- and down-regulation of gene expression in hippocampus by hypoxia compared to normoxia (H-N). Network inference and statistical modeling of gene ontology terms were performed similarly as reported for hypothalamus in Panel A.

**Figure 2:**
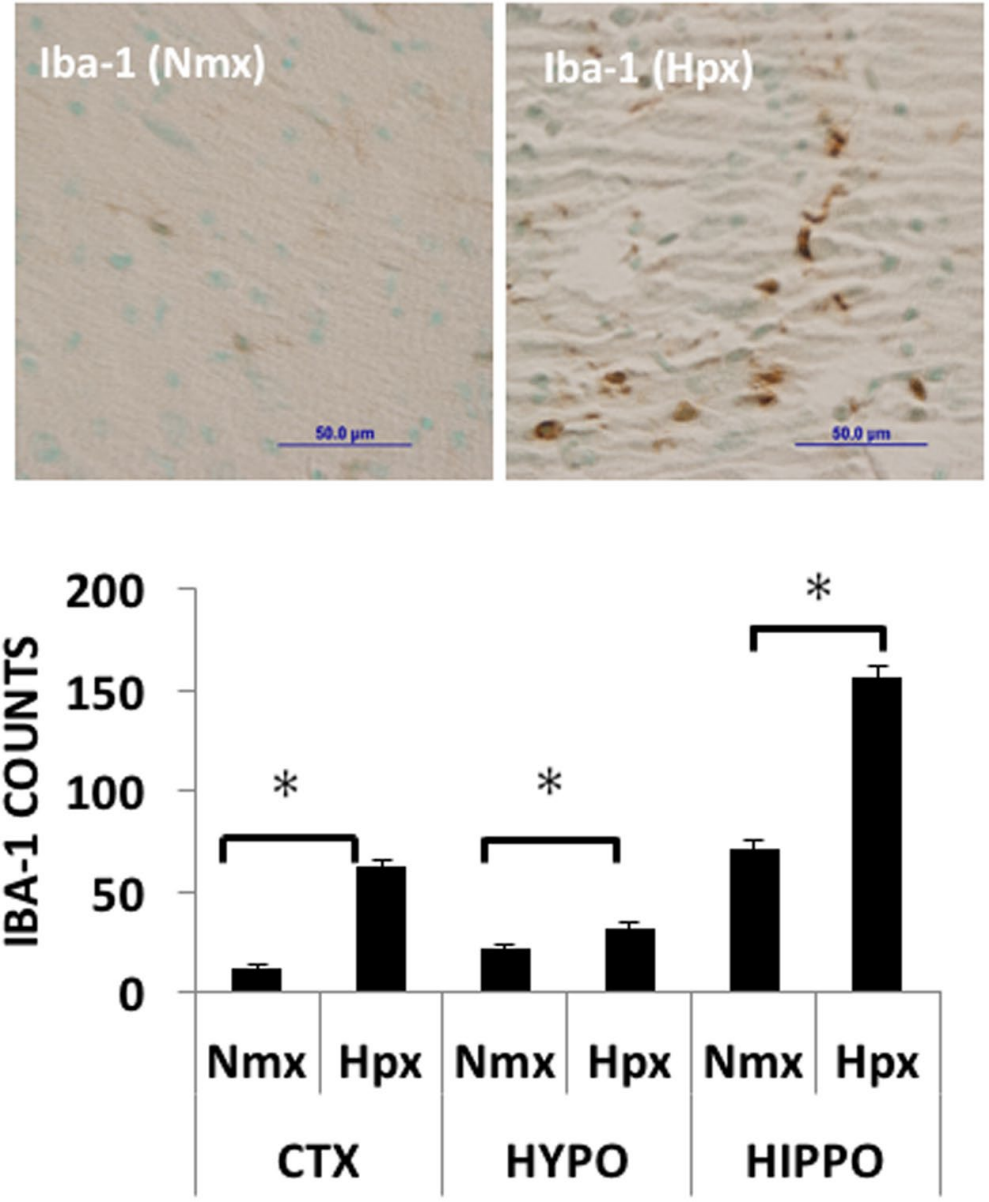
Representative micrographs from hypothalamus and numerical results of immunohistochemical analysis of cells expressing Iba-1 immunoreactivity in the fetal cerebral cortex (CTX, previously reported^4^), hippocampus (HIPPO, average measurements from regions CA1, CA2, CA3, CA4, and Dentate Gyrus), and hypothalamus (HYPO). Macrophage counts per field at 40X in the hippocampus and hypothalamus were averaged from 7 images per animal (in 5 subregions in hippocampus) and 3–4 animals per group. Data are expressed as mean ± SEM. Different letters indicate statistically significant difference. Scale bars 50 µm. Abbreviations: nmx, normoxia; hypx, hypoxia. *p < 0.05.

In the hypothalamus, 357 and 280 genes were down- and up-regulated, respectively, 24 h after a 30 min period of hypoxia, compared to the normoxic control (Fig. 1). In the hippocampus, 240 and 270 genes were down- and up-regulated, respectively, after hypoxia (Fig. 2). The biological processes associated with genes upregulated by hypoxia included lipopolysaccharide response and binding, cytokine-mediated signaling pathway, positive regulation of cell migration in hypothalamus, and innate immune response, cytokine stimulus response, and lipid response in hippocampus (Tables S2, S3). Molecular functions included interleukin-6 binding activity, chemokine receptor binding, glucocorticoid receptor binding in hypothalamus, and lipopolysaccharide binding, cytokine receptor activity, and glucose transmembrane transporter in the hippocampus (Tables S4, S5). Significant associations were revealed with MAPK signaling pathways, cytokine-cytokine receptor interaction, hematopoietic cell lineage, and toll-like receptor signaling pathway in hypothalamus, and cytokine-cytokine receptor interaction, hematopoietic cell lineage, and chemokine signaling pathway in hippocampus (Tables S6, S7).

The genes downregulated with hypoxia revealed no significant functions in hypothalamus, or cellular macromolecule metabolic process in hippocampus (Tables S2, S3). Significant molecular functions included protein binding in hypothalamus, and calcium transporting ATPase activity, alpha-amino-3-hydroxy-5-methyl-4-isoxazole propionate (AMPA) receptor activity, prostaglandin endoperoxide synthase activity, and enzyme, protein, RNA, and tubulin binding in hippocampus (Tables S4, S5). KEGG pathways significantly associated with downregulated genes included metabolic pathways, steroid biosynthesis, dorso-ventral axis formation, and notch signaling in hypothalamus, and cancer, spliceosome, protein processing in endoplasmic reticulum, p53 signaling, and long-term potentiation in hippocampus (Tables S6, S7).

Given the above associations, the relationship between hypoxia and inflammation was explored using qPCR, including changes in expression of other genes that were not on the array, but would be predicted to also be increased if inflammatory pathways were activated. Hypoxia increased gene expression in inflammation-related pathways in hypothalamus including that of TLR2, CD14, NFKBIA, IL1B, CXCL10, PTGS2, and CASP8 (Fig. S1). Similarly, in hippocampus, expression of TLR2, TLR4, NFKB, MYD88, CXCL10, and CASP8 increased (Fig. S2). Apoptosis-related pathways (e.g., NIK, AKT, APAF1, BAX, BAD, CASP3) were not upregulated.

## Evidence of increased microglia/macrophage abundance in fetal brain

While it was not surprising to find signs of inflammation after hypoxia, it was surprising that the pathways included toll-like receptors, TLR2 and TLR4. This suggested the presence of microglia or macrophages. To test the possibility that infiltration of the hypothalamus and hippocampus with microglia or macrophages might be causing this inflammation, sections of hypothalamus and hippocampus were immunostained for Iba-1, a marker of microglia and macrophages. Hypoxia increased the number of iba-1 positive cells in both hypothalamus and hippocampus (Fig. 2). This result parallels that previously found in cerebral cortex^4^ and kidney cortex^7^. Immunostaining for the adrenergic receptor P2Y_12_, a more specific marker for microglia^8^, revealed increases in P2Y_12_-positive cells in hypoxia compared to normoxia (normoxia vs hypoxia: 8.3 ± 1.4 vs 16.8 ± 2.1 and 29.5 ± 2.7 vs 40.7 ± 3.7 cells/field in cortex and hippocampus, respectively). These changes were statistically significant (p = 0.0006 and 0.013, respectively).

## Evidence for bacteria in fetal brain

**Figure 3:**
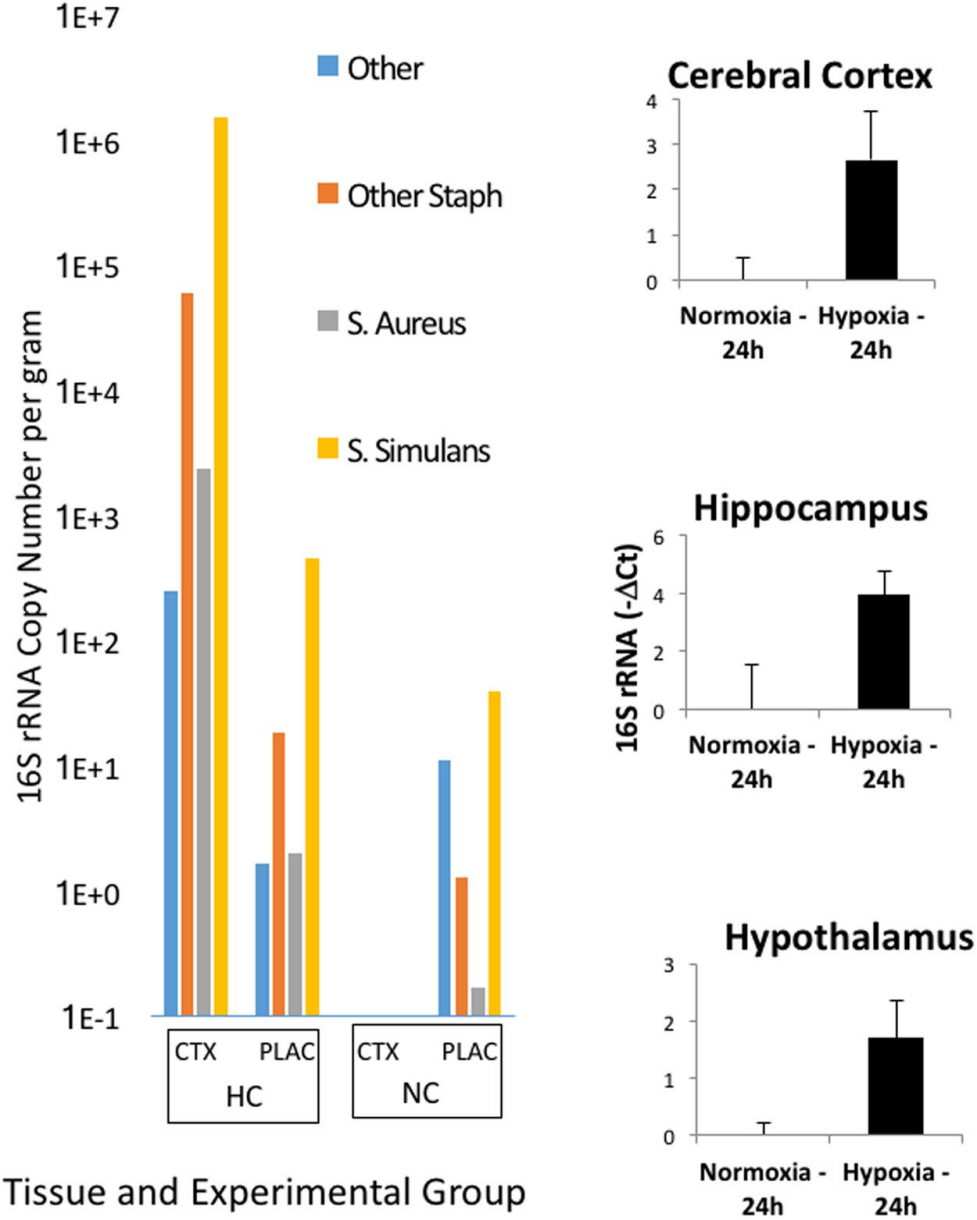
*Left*, Gene copy number of the 16S rRNA gene in bacterial species as identified by Sanger sequencing of DNA isolated from fetal cerebral cortex and placenta in fetuses exposed to hypoxia (HC) or normoxia (NC). Data are reported as distribution of total numbers of 16S rRNA genes per gram tissue wet weight in 4 fetuses in each of 4 groupings of bacterial genus and species (*S. simulans*, *S. Aureus*, other *Staphyloccocus*, and non-*Staphylococcus*). *Right*, qPCR analysis of 16S rRNA abundance in mRNA extracted from fetal hippocampus, hypothalamus, and cerebral cortex 24 h after transient hypoxia or normoxia.

To test whether the fetal brain inflammation was accompanied by a bacterial invasion, gram staining was performed as well as qPCR of 16S rRNA using bacterial universal primers that do not amplify eukaryotic genes. The hypothalamus, hippocampus, and cerebral cortex of hypoxia-exposed fetuses were all found to have significantly more bacterial 16S rRNA 24 hours after transient hypoxia compared to the normoxic control (Fig. 3). As one potential route for entry into the fetal brain, hypoxia increased vascular permeability as measured by the effusion of albumin from the vascular space to the interstitial space (Fig. S3).

**Figure 4:**
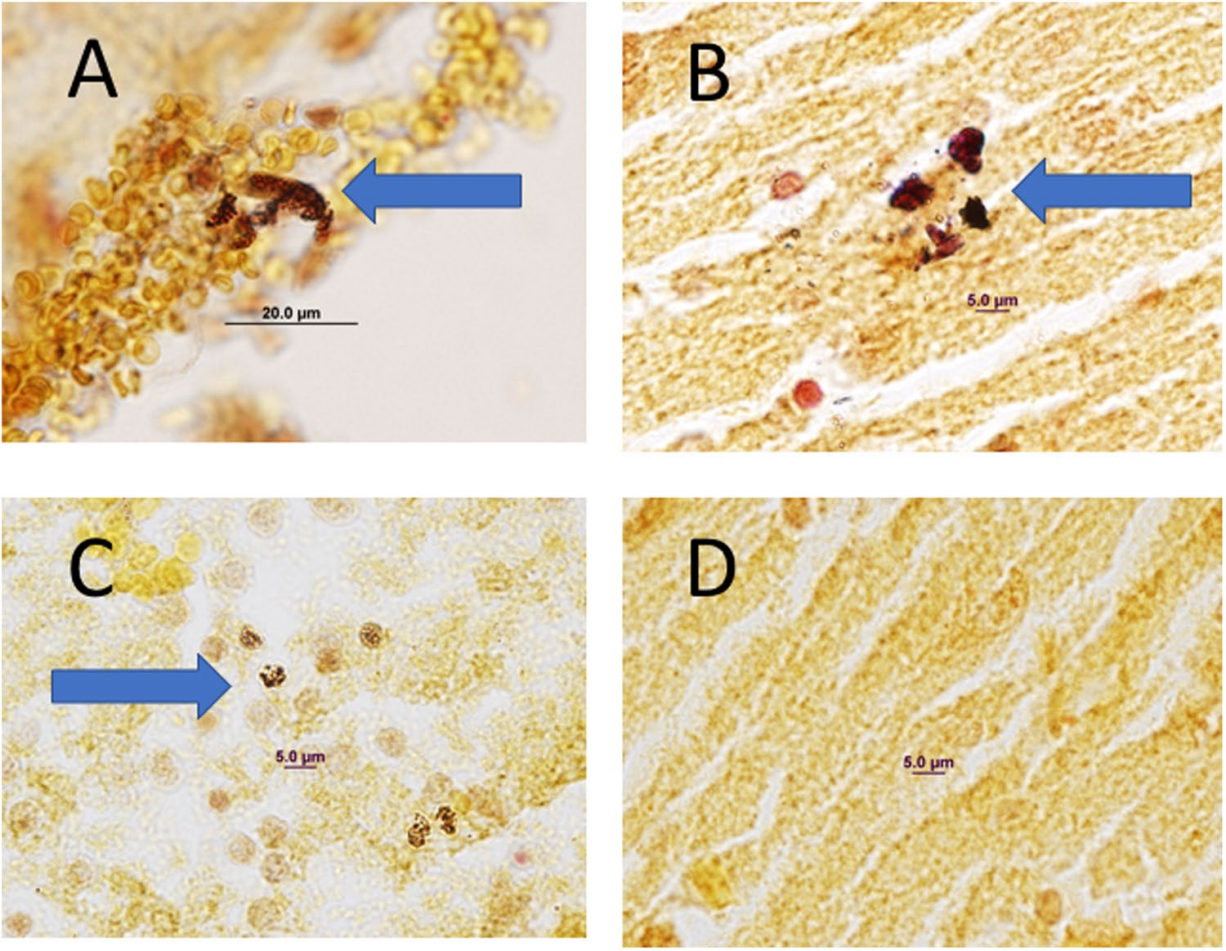
Gram stain of histological sections of cerebral cortex from hypoxia- and normoxia- exposed fetal sheep. Panel A, extravascular macrophage containing gram-positive inclusions with morphology similar to S. simulans. Panel B, gram-positive aggregates. Panel C, intracellular gram-positive bacteria, Panel D, gram stain of cerebral cortex (100x) from fetus exposed to normoxia.

Analysis of the relative abundance of bacterial taxa as represented in 16S rRNA and on the number of 16S rRNA gene cop ies in each sample showed that *Staphylococcus simulans* was the most common species in the cerebral cortex (Fig. 3). The abundance of bacteria in the fetal cerebral cortex of normoxic fetuses was much lower, but not absent. Histological analysis of fetal cerebral cortex, hippocampus, and hypothalamus revealed the presence of gram-positive bacteria in both control (normoxia) and hypoxia-exposed animals, although the abundance was very low in the animals from the normoxia group. Bacteria with morphology consistent with Staphylococcus were observed in extravascular macrophages, especially in the sub-pial regions (Fig. 4). Bacteria present in the brain parenchyma appeared to be clumped and many were clearly intracellular within cells that were similar in size and shape to activated microglia (Fig. 4).

## Presence of bacteria in placenta

**Figure 5:**
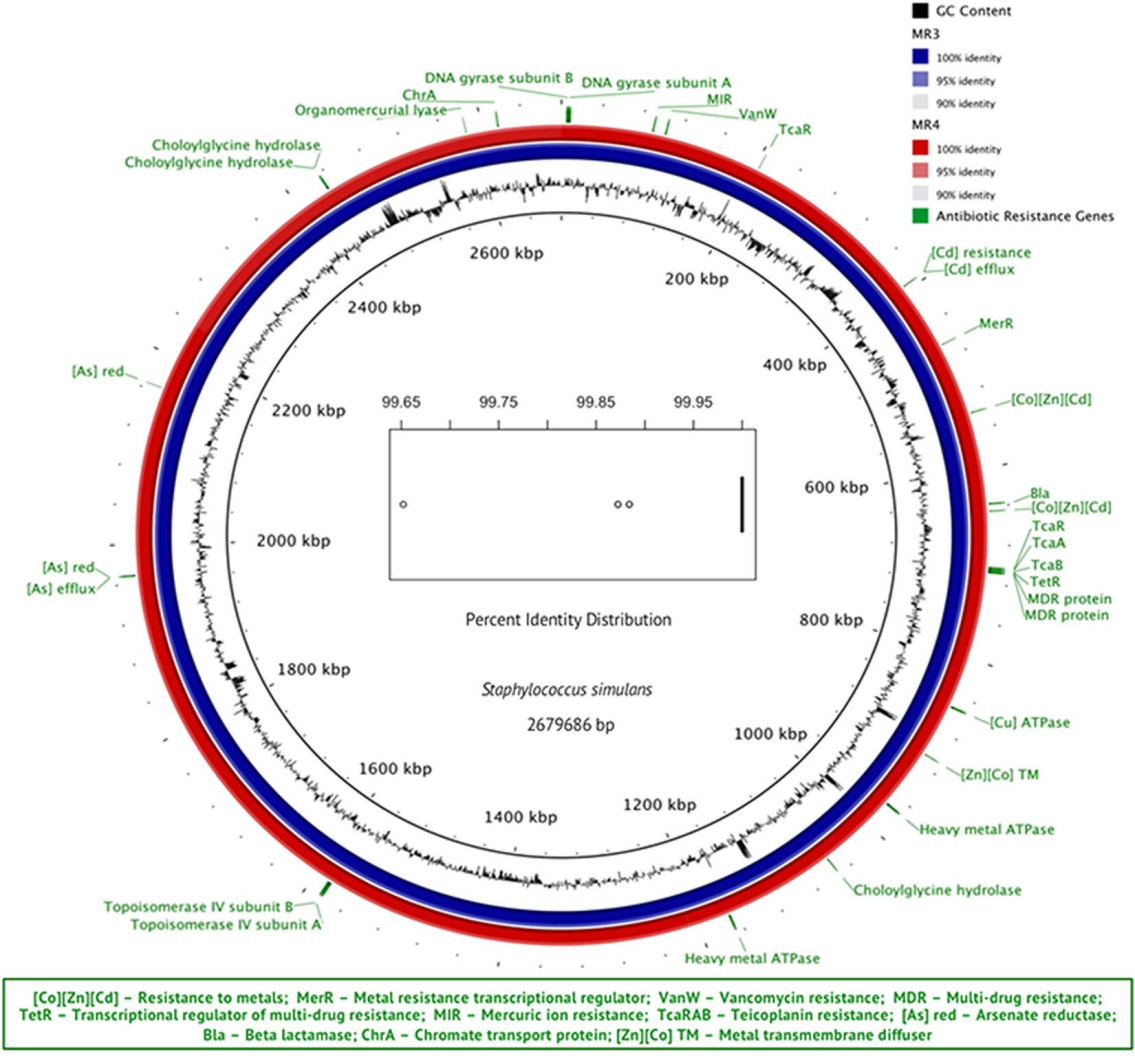
Circular map alignment of the genomes from S. simulans strains MR3 (from brain) and MR4 (from placenta). Alignment identity calculations were performed using the nucleotide BLAST algorithm implemented through BRIG. The inner black ring represents GC content for the sequences. Percent identity between the sequences is color coded at three percentage levels as shown in the legend (red for MR3, blue for MR4). Antibiotic resistance genes and their locations, as predicted by RAST, surround the map in green and are consistent between the genomes. Within the graphic is the average nucleotide identity between the genomes. Sequence identity is displayed as both a percent and a bit-score distribution. Calculations were performed in 1 kb windows, both one and two way, with a 95% identity threshold.

**Figure 6:**
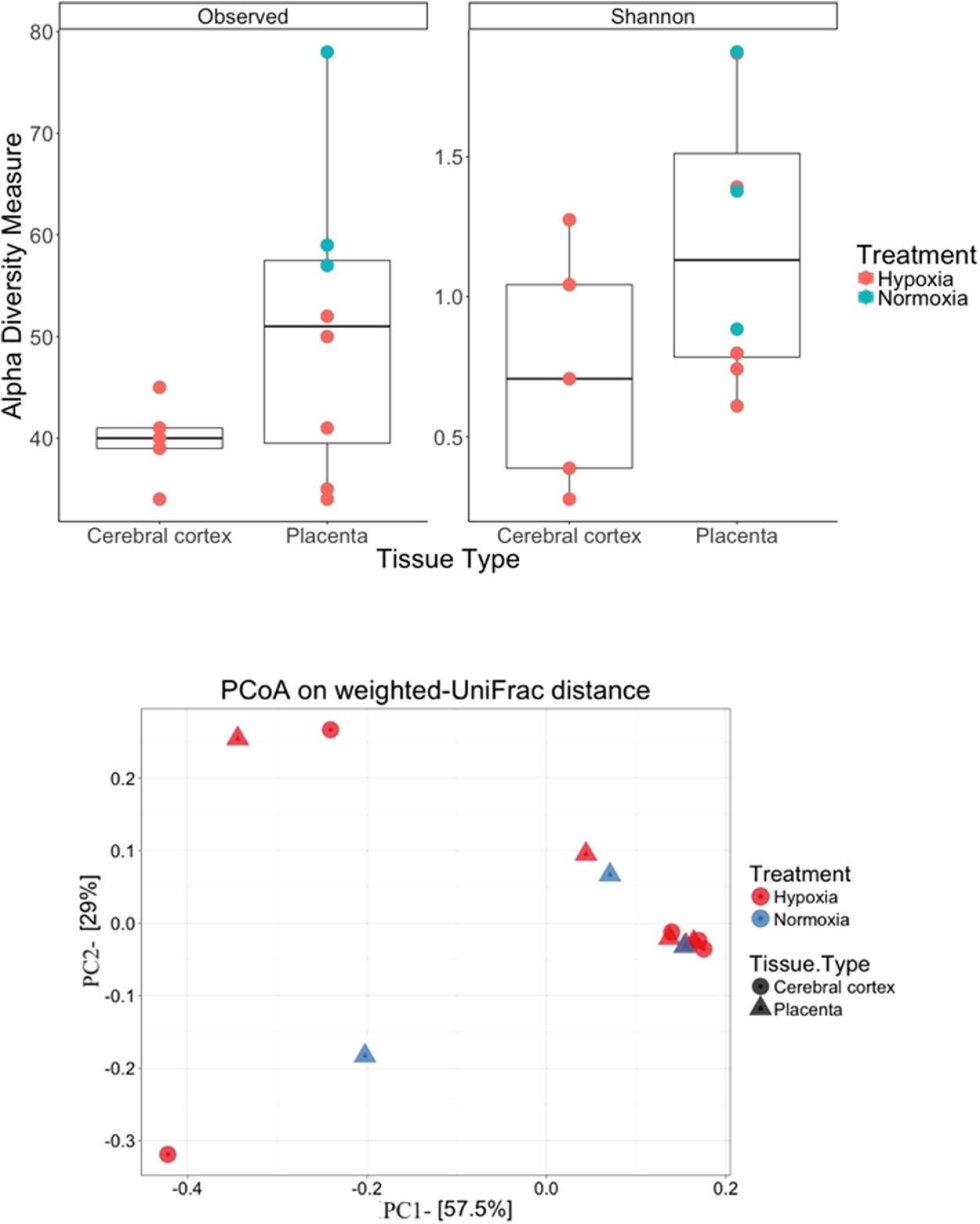
Alpha (Panel A) and Beta (Panel B) diversity of bacteria identified in the placenta and brain samples after amplification and sequencing of 16S rRNA genes from extracted DNA. Alpha diversity describes the bacterial diversity within each sample and is expressed as the number of operational taxonomic units defined by 95% identity of the 16S rRNA gene (observed - left panel) or as the Shannon diversity index (Shannon - right panel). Beta diversity compares the relationship of the community diversity between samples and is depicted as a principal component analysis plot (PCoA) using the weighted UniFrac distance measure. Bacterial diversity in cerebral cortex and placenta of fetal sheep exposed to normoxia or hypoxia.

*Staphylococcus simulans* was also most abundant in placentas of both normoxic and hypoxic ewes. To test for similarity of cortical and placental populations of bacteria, *Staphylococcus simulans* strains were cultured from a hypoxia-exposed placenta and fetal cerebral cortex and tested for antibiotic resistance. Strains from both locations were coagulase-negative and found to be equally resistant to ampicillin and kanamycin (both at 500 μg/ml), vancomycin (250 μg/ml), ciprofloxacin (100 μg/ml), chloramphenicol, erythromycin, gentamicin, oxacillin, rifampicin, spectinomycin, tetracycline, trimethoprim (all at 50 μg/ml), and methicillin (30 μg/ml). The genomes of *S. simulans* cultures from the placenta and fetal cerebral cortex of a hypoxia-treated ewe were identical at the nucleotide level (Fig. 5), suggesting that a common source. Alpha and beta diversity of the bacteria in brain and placenta were calculated for hypoxic and normoxic treatments (Fig. 6). The normoxic placental samples possessed a significantly higher number of bacterial taxa than did the hypoxic placental samples. No bacteria were observed in the cerebral cortex of the normoxic samples. In contrast, bacteria were observed in the hypoxic cerebral brain tissues and the number of taxa was considerably lower than in the hypoxic placenta.

## Discussion

The fetus is traditionally considered sterile^9^. In many cases of preterm birth, there is invasion of bacteria into the fetus, thought to be the result of an ascending vaginal infection^10^. A diverse bacterial population has been reported in the human placenta that more closely matches the diversity found in the human mouth^11^. In newborn infants, bacterial DNA is found in meconium (first stool)^12^, especially among preterm infants. The present study in sheep indicates that bacteria can become mobilized to the fetal brain as a consequence of hypoxic episodes. These results represent a sea change in our understanding of the presence of a microbiome in the fetus; the data strongly suggests that hypoxic stress

led to invasion of the fetal brain by *S. simulans* from the placenta. Preterm reterm neonates are especially vulnerable to polymicrobial bloodstream infections, 70% of which arise from coagulase negative *Staphylococcus* ^13^. Prior to birth, the neonatal adaptive immune response lacks robustness towards bacterial invasion. Based on our findings in preterm fetal sheep exposed to hypoxia, it is probable that the high abundance of *S. simulans* in both the cerebral cortex and placenta is directly correlated with a transiently suppressed or insufficient host immunological response after hypoxia. There are no reports of *S. simulans* crossing the blood-brain barrier but such reports do exist for *S. aureus* ^14, 15^. Regardless, the late gestation fetal blood brain barrier is likely to be far more permeable than that from a young lamb^16^. Our data suggest that maternal and/or fetal hypoxic stress mobilizes bacteria from the placenta or other tissue, allowing entry into into the fetal brain. Both maternal cells (maternal microchimerism) and virus transit the placenta^17, 18^; hypoxia may increase the transit rate of bacteria and/or increase delivery of bacteria to the brain as a consequence of the increased brain blood flow, as well as the increased permeability of the brain^19^. The high level of antibiotic resistance from the cultured *S. simulans* strains may be the result of selection following five days of ampicillin treatment of the ewe prior to the study. Future experiments are needed to confirm the source of these bacteria and the identity of the exposures that led to antibiotic resistance in these bacteria. In addition, it is not known whether maternal stresses other than hypoxia can lead to *Staphylococcus* invasion into the fetus, or if these events would lead to premature birth in sheep or humans. Commensurate populations of bacteria on maternal skin or in maternal mouth, vagina, mammary glands, or gastrointestinal tract may comprise a background of exposure of the pregnancy to bacteria. Maternal stress – which causes an increase in maternal cortisol secretion – may transiently reduce the ability of the maternal immune system to clear the small numbers of bacterial cells that would otherwise be cleared rather than transit the placenta to the fetus. If this were true, the specific genus, species, and strain of bacteria found in the fetus would be informed by the maternal microbiome. In the present experiments, we found *Staphylococcus simulans*, a bacterium commonly found in mouth^20^, on skin^21, 22^, and in mammary gland^23^ of various species. One might expect that other species or populations of animals might respond to hypoxia similarly, but with other species of *Staphylococcus* or other taxa altogether, gain depending on the commensurate bacteria resident in the pregnant mother.

It is perhaps logical to assume that inflammation in the fetal brain is damaging, or that inflammation might have negative consequences with regard to programming of adult disease. Cortical white matter damage, for example, is common in infants suffering birth asphyxia^24^, and is assumed to result from direct cellular oxygen deprivation. In our study the relatively brief period of hypoxia was not associated with activation of apoptotic pathways, suggesting there is not rapid cellular damage due to the bacteria. Microglia and macrophages are also important participants in tissue development and maturation. For example, microglia are a critical part of maintaining the balance between cell division and cell death in the developing brain and loss of microglial function might be causative with regard to known conditions involving cellular overgrowth^25^. Thus, whether the biology of fetal brain inflammation has great relevance to the ultimate outcome of the offspring is not known. Our results suggest, however, not only that bacteria may access the brain after periods of hypoxia in late pregnancy, but that the bacteria might be influenced by maternal antibiotic administration. The study has implications for treatment of mothers and newborns. For example, does maternal antibiotic treatment increase the risk of antibiotic resistant bacteria in the placenta and the fetus? Is anti-inflammatory treatment the best treatment for newborns at risk for hypoxic ischemic CNS damage?

## Methods

Sixteen chronically catheterized singleton or twin ovine fetuses were studied between the gestational age of 122 ± 5 days (full term = 145–147 days). All experiments were approved by the University of Florida Animal Care and Use Committee. All laboratory procedures were performed in accordance with relevant guidelines and regulations.

After 24 h of fasting of the pregnant ewes, fetal surgery was performed on 116 ± 3 days of gestation as previously described^4^. After recovery from surgery, ewes and their fetuses were studied while conscious and freestanding in their pens with access to food. Experimental design was as previously described^4^. In this report, we present the results from the normoxic control (NC) and hypoxic control (HC) groups (n = 4/group). Blood gases and fetal cardiovascular and neuroendocrine responses to hypoxia were reported previously^5^.

Fetuses were sacrificed 24 h post hypoxic stress, and various fetal tissues were snap frozen and stored at −80 °C. At sacrifice, fetal brain was dissected, processed and immunostained for Iba-1 as described previously^4, 6, 9^ and for P2Y_12_ using antiserum HPA014518 (1:200)^8^. Microarray analysis was performed and analyzed as previously described^26^.

### Real-time qPCR

RNA was extracted as previously described^4^. The same mRNA samples used for the microarray was also used for qPCR validation. The primers for measurement of ovine gene expression were designed based on the known *Ovis aries* and *Bos taurus* genomes (Table S1) for Taqman or Sybr green chemistry (Thermo-Fisher Scientific, Waltham, MA). Bacterial 16S rRNA was measured using bacterial universal primers^27^ and 16S rRNA copy number determined using a standard curve of *S. simulans* DNA.

### DNA extraction, 16S rRNA gene amplification and sequencing and diversity analysis

Placental and cerebral cortex DNA was extracted using the DNEasy Blood & Tissue Kit (Qiagen) following the manufacturer’s guidelines. DNA was extracted and quantified spectrophotometrically, amplification of 16S rRNA genes, and barcoded sequencing of the resulting amplicons was done as described previously^12, 28^. Diversity analyses were calculated and plots designed using phyloseq in R^29^.

### Bacterial culturing and genome sequencing from brain and placental tissues

Tissues were mechanically homogenized in 300 μl of Brain Heart Infusion Broth (BHI, Sigma-Aldrich) for 30 seconds from which 10-fold serial dilutions were prepared and spread on BHI solid medium. Following incubation at 37 °C for 24 h, selected colonies were purified and their identity determined by amplifying the16S rRNA gene using 8 F and 1492 R primers followed by Sanger sequencing. Whole genomes from selected strains of *Staphylococcus simulans* were sequenced and using the PacBio RS II platform as described previously^30^. Genome annotation was performed using the NCBI Prokaryotic Annotation Pipeline^31^ after submission to GenBank. Whole genome sequence comparison and circular map generation was done using BRIG^32^. One and two-way average nucleotide identity was calculated using the method of Goris *et al*.^33^. The genome sequences and raw sequence data are archived in Genbank accession numbers CP017428 and SRR4340965, respectively for *S. simulans* strain MR3; and CP017430 and SRR4340966, respectively for *S. simulans* strain MR4.

### Bacterial diversity

Filtered reads were grouped into Operational Taxonomic Units (OTUs), which were then classified at the phylum level with “R” statistical software. Relative differences in clustering amongst placenta and cerebral cortex bacterial communities were calculated using the Bray-Curtis Index and were then graphed by Principal Component Analysis (PCoA) in order to illustrate beta diversity in the sample set. In this case, shorter distances between plotted points indicated an increase in similarity in terms of microbial composition, where UniFrac distances were calculated based on shared branches within the phylogenetic tree.

Alpha diversity (Shannon-Wiener Index) was calculated to estimate the species richness and biodiversity of bacterial communities in the fetal placenta and fetal cerebral cortex, while also taking into account treatment with either hypoxia or normoxia. Overall, our results mirror the microbial colonization patterns observed from 16S rRNA sequencing, transcriptomics, and qPCR analyses, lending evidence that there is higher diversity in placental samples and considerably lower diversity in the cerebral cortex, while also indicating higher abundance of bacterial species in normoxic samples.

### Antibiotic Resistance Screening of S. simulans isolate 622B

Sensitivity of *S. simulans* cultures from brain and placenta to ampicillin, methicillin, oxacillin, kanamycin, vancomycin, tetracycline, rifampicin, gentamicin, chloramphenicol, ciprofloxacin, spectinomycin, trimethoprim, and erythromycin (Sigma Aldrich) was tested in BHI broth with antibiotics at various concentrations in 96-well plates. Viability of cells post-antibiotic treatment was tested by addition of 20 *μ*L PrestoBlue.

### Calculations and Statistical Analysis

Data are presented as mean values ± standard error of the mean (SEM). The ovine Agilent 15.5k array results were analyzed with R Studio’s Limma package for R software v.2.15.1, employing moderated t-test using empirical Bayes method for small sample size per group as previously described^34^. The qPCR data were analyzed Student’s t-test^35^. Gene networks were inferred using the GeneMania plugin of Cytoscape^36^. Gene functional annotation was analyzed using WEB-based GEne SeT AnaLysis Toolkit (WebGestalt)^37, 38^. In all tests, the criterion for statistical significance was p < 0.05.

### Data availability

The datasets generated during and/or analyzed during the current study are available in the NCBI Gene Expression Omnibus repository (GSE82016 and GSE97916).

## Electronic supplementary material

Supplementary Dataset 1
