# Supplementary material for: Post-hypoxia Invasion of the fetal brain by multidrug resistant Staphylococcus
Source: Sci Rep. 2017 Jul 25;7:6458. doi: 10.1038/s41598-017-06789-6 (PMC5527097; doi:10.1038/s41598-017-06789-6)
Supplement: Supplementary file 1 — Supplementary Dataset 1 [file 41598_2017_6789_MOESM1_ESM.doc]

Supplementary Material

**Post-hypoxia Invasion of the fetal brain by multidrug resistant *Staphylococcus***

Miguel A. Zarate^1^, Michelle Rodriguez^2^, Eileen I. Chang^1^, Jordan T. Russell^2^, Thomas J. Arndt^1^, Elaine M. Richards^3^, Beronica A. Ocasio^2^, Eva Aranda^2^, Elizabeth M. Gordon^1^, Kevin Yu^1^, Josef Neu^4^, Maureen Keller-Wood^3^, Eric W. Triplett^2^, and Charles E. Wood^1^

**Figure Legends: Supplementary Material**

**Supplementary Figure 1.** Gene expression (mRNA abundance) in fetal hypothalamus measured by real-time qPCR for genes in the toll-like receptor inflammation pathway. Values are represented as negative delta cycle threshold (-ΔCt) compared to the NC group. The criterion for statistical significance was *P*<0.05 (Student’s t-test). Data are presented as means±SEM, and the y-axis scale varies between plots. *, statistically significant difference compared to normoxia.

**Supplementary Figure 2.** Gene expression (mRNA abundance) in fetal hippocampus measured by real-time qPCR for genes in the toll-like receptor inflammation pathway. Values are represented as negative delta cycle threshold (-ΔCt) compared to the NC group. The criterion for statistical significance was *P*<0.05 (Student’s t-test). Data are presented as means±SEM, and the y-axis scale varies between plots. *, statistically significant difference compared to normoxia.

**Supplementary Figure 3.** Representative micrographs from cerebral cortex and numerical results of immunohistochemical analysis from fetal hypothalamus and cerebral cortex of albumin leakage from microvasculature. Number of broken blood vessels were counted in each 40X field in the hypothalamus (HYPO) and cerebral cortex (CTX) and averaged from 7 images per animal and 3-4 animals per group. Data are expressed as mean ± SEM. Different letters indicate statistically significant difference. Scale bars 50 µm. Abbreviations: Nmx, normoxia; Hypx, hypoxia.


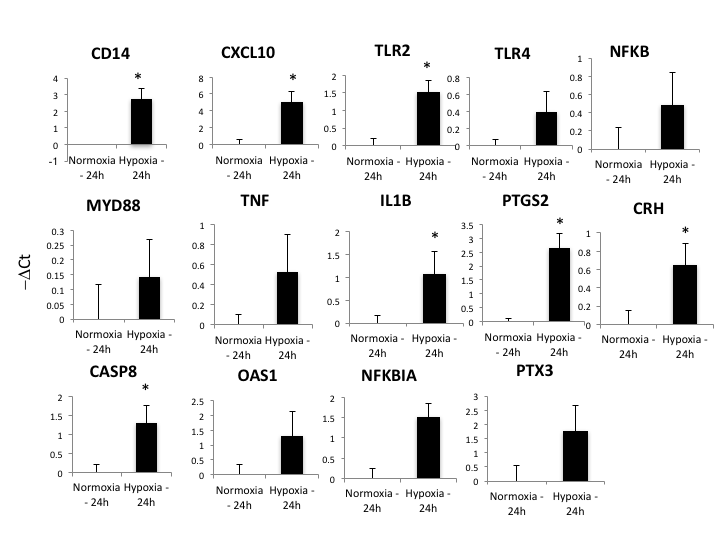


Supplementary Figure 1.

Supplementary Figure 2.

Supplementary Figure 3.

Supplementary Table 1. Primers and probes used for qPCR analysis (all sequences 5’-3’).

| gene | forward primer | reverse primer | probe |
| --- | --- | --- | --- |
| ACTB | TTCCTTCCTGGGCATGGA | GACGTCACACTTCATGATGGAATT | TCCTGCGGCATTCACGAAACTACCTT |
| CASP8 | TGGCTGCCCTCAAGTTCCT | GGAATAGCATCAAGGCATCCTT |  |
| CD14 | CCTAAAGGACTGCCGACCAA | GCGGCTCCCTGCTTAGCT |  |
| CRH | TCCCATTTCCCTGGATCTCA | GAGCTTGCTGCGCTAACTGA | TTCCACCTCCTCCGAGAAGTCTTGGAAAT |
| CXCL10 | TTGAACTGATTCCTGCAAGTCA | TTCCTTTTCATTGTGGCAATAATCT |  |
| IL1B | CGTGGCCATGGAGAAGCT | GGTCATCATCACGGAAGACATGT |  |
| MYD88 | GCCTGAGTATTTTGATGCCTTCA | GCTGCCGGATCATCTCATG |  |
| NFKB1 | TCCCACAGATGTTCACAAACAGT | GACGCTCAATCTTCATCTTGTGAT |  |
| NFKBIA | CTACACCTTGCCTGTGAGCA | AGACACGTGTGGCCATTGTA |  |
| OAS1 | GAGGAAAGAGGGCGAGTTCT | GGATGAGGCTCTTCAGCTTG |  |
| PTGS2 | GCACAAATCTGATGTTTGCATTCT | CTGGTCCTCGTTCATATCTGCTT | TGCCCAGCACTTCACCCATCAATTTT |
| PTX3 | GCACCTGGGATTCAAAGAAA | TGTTTCATCAAAGCCACCAA |  |
| TLR2 | GATTCTGCTGGAGCCCATTG | TCATGATCTTCCGCAGCTTACA |  |
| TLR4 | ACTCGCTCCGGATCCTAGACT | CCTTGGCAAATTCCGTAGTTCT |  |
| TNF | CCCTTCCACCCCCTTGTT | ATGTTGACCTTGGTCTGGTAGGA | |
| bacterial 16S rRNA | TCCTACGGGAGGCAGCAGT | GGACTACCAGGGTATCTAATCCTGTT | |

**Supplementary Table 2.** Top 10 gene ontology biological processes significantly regulated by hypoxia in the fetal hypothalamus.

| **Groups** | **Biological Processes** | **# Genes involved** | **Adjusted**  ***P*-values** |
| --- | --- | --- | --- |
| Hypoxia  *Up-regulated compared to normoxia* | Response to lipopolysaccharide | 28 | 2.83E-14 |
|  | Cytokine-mediated signaling pathway | 29 | 2.80E-10 |
|  | Positive regulation of cell migration | 24 | 2.41E-10 |
| Hypoxia  *Down-regulated compared to normoxia* | No significant biological processes |  |  |

**Supplementary Table 3.** Top 10 gene ontology biological processes significantly regulated by hypoxia in the fetal hippocampus..

| **Groups** | **Biological Processes** | **# Genes involved** | **Adjusted**  ***P*-values** |  | **Adjusted**  ***P*-values** |
| --- | --- | --- | --- | --- | --- |
| Hypoxia  *Up-regulated compared to normoxia* | innate immune response | 33 | 7.06E-09 |  | |
|  | response to cytokine stimulus | 28 | 1.79E-08 |  | |
|  | response to lipid | 34 | 1.08E-08 |  | |
|  | lipopolysaccharide binding | 34 | 1.02E-04 |  | |
| Hypoxia  *Down-regulated compared to normoxia* | regulation of cellular macromolecule metabolic process | 106 | 2.00E-04 |  |  |
|  | cellular macromolecule metabolic process | 153 | 1.00E-04 |  |  |

| **Groups** | **Molecular Functions** | **# Genes involved** | **Adjusted**  ***P*-values** |
| --- | --- | --- | --- |
| Hypoxia  *Up-regulated compared to normoxia* | Fibronectin binding | 6 | 1.00E-4 |
|  | Protease binding | 5 | 2.62E-4 |
|  | Glucocorticoid receptor binding | 3 | 8.60E-3 |
|  | Heparin binding | 9 | 8.6E-3 |
|  | Integrin binding | 7 | 8.6E-3 |
|  | Interleukin-6 binding activity | 2 | 8.6E-3 |
|  | Glycoprotein binding | 6 | 1.08E-2 |
|  | BH3 domain binding | 2 | 1.97E-2 |
|  | Ciliary neurotrophic factor receptor activity | 2 | 1.97E-2 |
|  | Chemokine receptor binding | 5 | 2.62E-2 |
| Hypoxia  *Down-regulated compared to normoxia* | Protein binding | 171 | 0.0274 |

**Supplementary Table 4.** Top 10 gene ontology molecular functions significantly regulated by hypoxia in the fetal hypothalamus

**Supplementary Table 5.** Top 10 gene ontology molecular functions significantly regulated by hypoxia in the fetal hippocampus.

| **Groups** | **Molecular Functions** | **# Genes involved** | **Adjusted**  ***P*-values** |
| --- | --- | --- | --- |
| Hypoxia  *Up-regulated compared to normoxia* | threonine-type peptidase activity | 4 | 1.11E-2 |
|  | protein-disulfide reductase activity | 2 | 1.11E-2 |
|  | cytokine receptor activity | 7 | 1.11E-2 |
|  | lipopolysaccharide binding | 3 | 2.22E-2 |
|  | glucose transmembrane transporter | 3 | 8.6E-3 |
| Hypoxia  *Down-regulated compared to normoxia* | Calcium transporting ATPase activity | 4 | 6.00E-04 |
|  | alpha-amino-3-hydroxy-5-methyl-4-isoxazole propionate selective glutamate receptor activity | 3 | 1.10E-3 |
|  | enzyme binding | 37 | 1.10E-03 |
|  | protein domain specific binding | 21 | 9.90E-03 |
|  | prostaglandin endoperoxide synthase activity | 2 | 1.27E-02 |
|  | RNA binding | 26 | 1.49E-02 |
|  | tubulin binding | 9 | 3.47E-02 |

| **Groups** | **KEGG Pathways** | **# Genes involved** | **Adjusted**  ***P*-values** |
| --- | --- | --- | --- |
| Hypoxia  *Up-regulated compared to normoxia* | Osteoclast differentiation | 16 | 9.39E-14 |
|  | MAPK signaling pathway | 17 | 4.43E-10 |
|  | Cytokine-cytokine receptor interaction | 16 | 2.55E-09 |
|  | Pathways in cancer | 17 | 4.74E-09 |
|  | Amoebiasis | 11 | 4.85E-09 |
|  | Malaria | 8 | 4.22E-08 |
|  | Hematopoietic cell lineage | 9 | 1.76E-07 |
|  | Toxoplasmosis | 10 | 4.33E-07 |
|  | Toll-like receptor signaling pathway | 9 | 5.04E-07 |
|  | Chagas disease | 9 | 5.37E-07 |
| Hypoxia  *Down-regulated compared to normoxia* | Metabolic pathways | 30 | 2.09E-06 |
|  | Steroid biosynthesis | 4 | 0.0007 |
|  | Dorso-ventral axis formation | 4 | 0.0012 |
|  | Pathways in cancer | 10 | 0.0067 |
|  | Bladder cancer | 4 | 0.0067 |
|  | Notch signaling pathway | 4 | 0.0084 |
|  | Endocytosis | 7 | 0.0180 |
|  | PPAR signaling pathway | 4 | 0.0252 |
|  | Renal cell carcinoma | 4 | 0.0252 |
|  | Ether lipid metabolism | 3 | 0.0260 |

**Supplementary Table 6**. Top 10 enriched KEGG pathways that were significantly regulated in the fetal hypothalamus.

**Supplementary Table 7.** Top 10 enriched KEGG pathways that were significantly regulated in the fetal hippocampus.

| **Groups** | **KEGG Pathways** | **# Genes involved** | **Adjusted**  ***P*-values** |
| --- | --- | --- | --- |
| Hypoxia  *Up-regulated compared to normoxia* | Cytokine-cytokine receptor interaction | 16 | 5.80E-10 |
|  | Hematopoietic cell lineage | 8 | 2.55E-6 |
|  | Osteoclast differentiation | 8 | 3.10E-5 |
|  | Adipocytokine signaling pathway | 6 | 5.83E-5 |
|  | Chagas disease (American trypanosomiasis) | 7 | 5.83E-5 |
|  | Proteasome | 5 | 9.4E-5 |
|  | Malaria | 5 | 0.0002 |
|  | Pathways in cancer | 10 | 0.0002 |
|  | Chemokine signaling pathway | 8 | 0.0002 |
|  | Metabolic pathways | 20 | 0.0002 |
| Hypoxia  *Down-regulated compared to normoxia* | Pathways in cancer | 11 | 0.0006 |
|  | Spliceosome | 7 | 0.0006 |
|  | Protein processing in endoplasmic reticulum | 8 | 0.0006 |
|  | Endocytosis | 8 | 0.0010 |
|  | p53 signaling pathway | 5 | 0.0010 |
|  | Long-term potentiation | 5 | 0.0010 |
|  | Prion diseases | 4 | 0.0010 |
|  | mRNA surveillance pathway | 5 | 0.0016 |
|  | Ribosome biogenesis in eukaryotes | 5 | 0.0016 |
|  | Amyotrophic lateral sclerosis (ALS) | 4 | 0.0029 |

1 Chang, E. *I. et a*l. Ketamine decreases inflammatory and immune pathways after transient hypoxia in late gestation fetal cerebral cortex*. Physiol R*e**p** 4, doi:10.14814/phy2.12741 (2016).
